# Association of Beck Depression Inventory score and Temperament and Character Inventory-125 in patients with eating disorders and severe malnutrition

**DOI:** 10.1186/s40337-015-0077-8

**Published:** 2015-11-05

**Authors:** Satoshi Tanaka, Keizo Yoshida, Hiroto Katayama, Kunihiro Kohmura, Naoko Kawano, Miho Imaeda, Saki Kato, Masahiko Ando, Branko Aleksic, Kazuo Nishioka, Norio Ozaki

**Affiliations:** Department of Psychiatry, Nagoya University Hospital, 65 Tsurumai, Showa, Nagoya, Aichi 466-8550 Japan; Health Care Promotion Division, DENSO Corporation, 1-1, Showacho, Kariya, Aichi 448-8661 Japan; Minami-Seikyo Hospital, 2-204, Minamioodaka, Midori, Nagoya, Aichi 459-8540 Japan; Seichiryo Hospital, 16-27, Tsurumai 4, Showa, Nagoya, Aichi 466-0064 Japan; Institute of Innovation for Future Society, Nagoya University, Furocho, Chikusa, Nagoya, Aichi 464-8601 Japan; Department of Psychiatry, Nagoya University Graduate School of Medicine, 65 Tsurumai, Showa, Nagoya, Aichi 466-8550 Japan; Saku Central Hospital, 197 Usuda, Saku, Nagano 384-0301 Japan; Center for Advanced Medicine and Clinical Research, Nagoya University Hospital, 65 Tsurumai, Showa, Nagoya, Aichi 466-8550 Japan

**Keywords:** Anorexia nervosa, Depression, Eating disorder, Harm avoidance, Self-directedness, Severe malnutrition

## Abstract

The authors investigated the association between personality and physical/mental status in malnourished patients with eating disorders. A total of 45 patients with anorexia nervosa, avoidant/restrictive food intake disorder, and other specified feeding or eating disorders were included and compared with 39 healthy controls. Personality characteristics and severity of depression were assessed using the Temperament and Character Inventory-125 and Beck’s Depression Inventory. Depression correlated with harm avoidance and self-directedness in both cases and controls. Body mass index did not correlate with personality in either group. These findings should be verified by longitudinal studies with higher weight/weight recovered patients.

## Introduction

Anorexia nervosa (AN) is a rare but debilitating eating disorder (ED) that affects 0.7 % of teenage females [1, 2]. A review on the prognosis of AN in the 20th century showed a mortality rate of 5.0 %. In addition, vomiting, bulimia, and purgative abuse are associated with poor outcomes and chronicity of the illness [3]. For severe and enduring cases, evidence of efficacy of treatment approaches is limited, and one review concluded that treatment trials need to move beyond targeting the core eating disorder pathology [4].

A malnourished state is also seen in patients diagnosed with other eating disorders. The Diagnostic and Statistical Manual of Mental Disorders, 5th edition (DSM-5) [5] has generated new diagnostic classifications including avoidant/restrictive food intake disorder (ARFID) and other specified feeding or eating disorder (OSFED). ARFID patients refuse adequate nourishment, but are not accompanied by AN-like psychopathology. OSFED patients do not fully meet the criteria of other specific eating disorders. These two diagnoses were formerly included in eating disorder, not otherwise specified (EDNOS) in DSM-IV-TR [6].

The personality of patients with AN has been examined in many studies with the use of a number of methods to measure personality. Among them, the Temperament and Character Inventory (TCI) theory [7] has been a lot utilized [8–11], but the results are inconsistent (Table 1). Few transdiagnostic studies have examined patients with EDs with malnutrition, including EDNOS [8].

**Table 1:** Past studies utilizing TCI to measure personality of AN patients: comparison with healthy controls

Participants	n	NS	HA	RD	PS	SD	CO	ST
AN [8]	122	n.s.	↑	n.s.	n.s.	↓	↑	n.s.
AN [9]	141	↓	↑	n.s.	n.s.	↓	n.s.	n.s.
AN-R [10]^a^	66	↓	n.s.	↓	↑	n.s.	n.s.	↓
AN-R [9]	70	n.s.	↑	n.s.	↑	↓	n.s.	n.s.
AN-R [11]	146	↓	↑	↓	↑	n.s.	↓	↓
AN-BP [10]^a^	59	n.s.	n.s.	↓	↑	n.s.	n.s.	↓
AN-BP [9]	71	n.s.	↑	n.s.	n.s.	↓	n.s.	n.s.
AN-BP [11]	60	n.s.	↑	n.s.	n.s.	↓	↓	n.s.

*TCI* Temperament and Character Inventory, *AN* anorexia nervosa, *n* number, *AN-R* anorexia nervosa, restricting type, *AN-BP* anorexia nervosa binge-eating/purging type, *NS* novelty seeking, *HA* harm avoidance, *RD* reward dependence, *PS* persistence, *SD* self-directedness, *CO* cooperativeness, *ST* self-transcendence, ↑ significantly higher than healthy controls, ↓ significantly lower than healthy controls, *n.s.* not significant

^a^Data adjusted for Beck Depression Inventory scale and age

A previous study reported that in patients with major depression, the Hamilton Rating Scale for Depression [12] correlates positively with Harm Avoidance (HA) and negatively with Self-Directedness (SD) and Cooperativeness (CO) [13] on TCI. From other reports, in patients with postpartum depression, HA increases longitudinally and correlates positively with the Edinburgh Postnatal Depression Scale [14–16]. These findings present the possibility that one’s personality measured via the TCI could be modulated by mental states, and this association could explain the inconsistency among TCI findings in patients with EDs (Table 1). We assumed that the personality of malnourished patients would be associated with their mental and physical states and sub-diagnosis, and used the TCI-125 [7, 17] to assess the personality of these patients and determine: 1) if the subscales of the TCI, especially HA, SD, and CO, are associated with findings on Beck’s Depression Inventory (BDI) [18, 19], and 2) if the TCI subscales can be associated with malnourishment, as assessed by body mass index (BMI).

### Participants and methods

All participants were Japanese women living in Japan. Among patients who met the criteria for ED of DSM-IV-TR, we excluded participants who were diagnosed with bulimia nervosa, younger than 17 years, or who did not consent to study participation. We recruited 46 patients with EDs (13 with AN, restricting type [AN-R], 22 with AN, binge-eating/purging type [AN-BP], 11 with EDNOS by DSM-IV-TR) from inpatients at Nagoya University Hospital, but we could not acquire enough life history from one patient with AN-BP, so only 45 cases were included in this study.

The 45 patients were retrospectively re-diagnosed by DSM-5 as follows: 14 patients with AN-R (13 formerly diagnosed as AN-R and one as EDNOS), 22 patients with AN-BP (21 formerly diagnosed as AN-BP and one as EDNOS), 4 patients with ARFID (formerly diagnosed as EDNOS), and 5 patients with OSFED (formerly diagnosed as EDNOS).

The BMI of patients ranged from 10.34 to 16.13 kg/m^2^. The BMI of 40 cases was less than 15 kg/m^2^, so most patients were diagnosed as severely malnourished [5]. They were treated with medication, medical nutrition, and supportive psychotherapy and behavioral therapy during hospitalization. Control participants, recruited from medical students and hospital staff, were all female. After a physical examination, they were interviewed to confirm the absence of psychiatric disorders.

Two questionnaires were used in this study: the Japanese version of the BDI [18, 19] and the Japanese version of the TCI-125 [17]. The Japanese version of the BDI was translated by Hasama et al. [18], and the TCI-125 was translated by Kijima et al. [17]; both translated versions were similarly reliable and had a factor-validity similar to that of the original version.

The ethics review committees at Nagoya University Graduate School of Medicine and Nagoya University Hospital approved the study protocol, and written, informed consent was obtained from all participants.

### Statistical analysis

All statistical analyses were performed using SPSS-23 software (SPSS Inc., Chicago, IL, USA). Demographics of participants were compared between cases and controls using the unpaired student *t*-test (two-tailed). *P* < 0.05 was regarded as significant. Total BDI scores and subscales of the TCI-125 were compared between cases and controls using the unpaired student *t*-test (two-tailed). P values were corrected using Bonferroni’s method, and final statistical significance was set at 0.05/8 = 0.0063. Power analysis was performed with G*Power 3 [20]. The above analysis has a 80.0 % statistical power when a large effect size (*d* = 0.8), according to Cohen’s criteria [21], is assumed. Total BDI scores and subscales of the TCI-125 were also compared among participants with AN-R, AN-BP, ARFID, OSFED, and controls, using one-way factorial analysis of variance (two-tailed), followed by Tukey’s post-hoc tests. *p* < 0.0063 was regarded as significant. The above analysis has a 99.9 % statistical power when a large effect size (*d* = 0.8) is assumed. Pearson product–moment correlation coefficients (*r*) were calculated between TCI subscales and BMI in addition to BDI on the cases and the controls respectively. Alpha was set at 0.05 and then corrected using Bonferroni’s method and finally set at 0.05 / 7 = 0.0071. Correlations between BMI and BDI were also analyzed (alpha was set at 0.05).

## Results

The BMI of cases was significantly lower than that of controls; no significant differences in age were seen, but education years of cases were significantly lower than that of controls (Table 2). BDI, HA, and Persistence (PS) on TCI-125 were significantly higher in cases, and SD was significantly lower in cases than in controls (Table 2).

**Table 2:** Demographics, BDI, and TCI-125 subscales of participants

		Cases (*n* = 45)	Controls (*n* = 39)	*p* value (student *t*-test)
Demographics				
	Age (years)	28.38 ± 8.76	27.67 ± 7.46	0.692
	BMI (kg/m^2^)	13.16 ± 1.42	21.71 ± 3.51	<0.001*
	Education (years)	13.96 ± 2.17	15.69 ± 1.67	<0.001*
BDI		23.07 ± 9.70	5.18 ± 5.03	<0.001**
TCI-125 subscales				
	NS	7.24 ± 3.53	8.56 ± 2.46	0.054
	HA	14.67 ± 4.30	11.79 ± 3.75	0.002**
	RD	9.82 ± 2.50	10.90 ± 2.20	0.04
	PS	3.24 ± 1.63	1.92 ± 1.55	<0.001**
	SD	11.73 ± 4.84	17.33 ± 4.47	<0.001**
	CO	17.96 ± 3.52	19.00 ± 2.10	0.11
	ST	3.84 ± 3.23	3.33 ± 2.74	0.44

Values are mean ± standard deviation. All variances are assumed to be equal

*BDI* Beck’s Depression Inventory, *TCI-125* Temperament and Character Inventory-125, *BMI* body mass index, *NS* novelty seeking, *HA* harm avoidance, *RD* reward dependence, *PS* persistence, *SD* self-directedness, *CO* cooperativeness, *ST* self-transcendence

*p < 0.05 is considered significant in comparisons of demographics

**p < 0.0063 is considered significant in comparisons of BDI and TCI-125 subscales

In the analysis of sub-diagnostic groups and controls, BDI was significantly higher in participants with AN-R, AN-BP, and OSFED than in controls (AN-R 22.07 ± 5.95 vs. controls 5.18 ± 5.03, *p* < 0.001; AN-BP 25.77 ± 10.45 vs. controls, *p* < 0.001; OSFED 19.80 ± 11.43 vs. controls, *p* = 0.001). PS was significantly higher in participants with AN-BP than in controls (3.59 ± 1.76 vs. 1.92 ± 1.55, *p* = 0.001), and SD was significantly lower in participants with AN-R (11.57 ± 4.20) and AN-BP (10.27 ± 4.75) than in controls (17.33 ± 4.47) (*p* = 0.001, *p* < 0.001, respectively).

No significant correlations were observed between any subscale of the TCI-125 and BMI in cases. A moderate positive relationship was observed both in cases (*r* = 0.47, *p* = 0.001) and controls (*r* = 0.52, *p* = 0.001) between BDI and HA, while a moderate negative relationship was observed between BDI and SD in cases (*r* = −0.50, *p* < 0.001) and controls (*r* = −0.69, *p* < 0.001). No significant correlations were observed between BMI and BDI both in cases or controls.

## Discussion

The present study revealed that BDI, HA, and PS on TCI-125 were significantly higher in cases, and SD was significantly lower in cases than in controls. In malnourished patients as well as in healthy controls, we found a significant relationship between TCI subscales and depression; in contrast, no relationship was seen between TCI subscales and BMI in either group.

AN patients are not always depressed [22–25] but frequently have depressive symptoms [26, 27] and have a tendency toward high PS and HA, and low Novelty Seeking (NS), Reward Dependence (RD) and SD (Table 1). These findings fit our current results largely considering AN-R and AN-BP, and are consistent with the clinical observation that our malnourished patients frequently show too much vigilance and/or avoidance of the practitioner’s treatment proposal even when AN-like drive for thinness or fear of gaining weight are absent. As for depression, antidepressants or antipsychotics for AN show no evidence of efficacy [28, 29]; therefore, the development of specific psychological approaches [30] is needed.

A review [26] indicated that the more the patient gained weight the less depressed he/she was, though no significant correlation was obtained between BMI and BDI in the present results. There is a possibility that patient characteristics of severe malnourishment in this study have confounded the results. This study also showed no significant relationship between TCI subscales and BMI. These findings suggest that gaining weight affects mood state, but not personality characteristics, though this study of cross-sectional design could not add much evidence. Klump et al. [8] reported that women with eating disorders in both the ill and recovered state show higher levels of HA and lower SD and CO scores than normal control women, indicative of trait-related personality characteristics. Malnourishment might have different effects on mood state and personality characteristics.

One of the strengths of this study is diagnostic application of the new DSM-5 criteria. Another strength is that most of the cases are severely malnourished (BMI < 15) and it is of clinical importance to investigate these difficult-to-treat patients specifically; however, this strength generates a weakness that the findings obtained in this study is difficult to be generalized.

One major limitation of this study is the relatively small sample size. The study is based on a cross-sectional model, and data sampling was not performed after weight recovery. In addition, neither the effect of nutrition therapy for depression nor possible personality changes were examined. Lack of the assessment of cognitive function might have confounded the results. Our study was limited to Japanese females living in Japan, and validation studies are needed in other cultural settings. Another limitation is related to the sampling biases, that is, cases were all inpatients undergoing re-nutritional therapy, which they do not always accept. The environment could lead to a decline in self-efficacy and affect the patients’ responses to questionnaires. In addition, informed consent was obtained in a strict manner, so only a highly cooperative group was sampled.

In order to fully apply the results obtained in this study into the clinical practice, it should be investigated whether the time-course of depression or malnourishment can affect personality characteristics in patients using longitudinal study design with higher weight/weight recovered patients. Furthermore, it should be confirmed that depression or malnourishment effect on personality characteristics is similar in patients and healthy controls.

## Abbreviations

AN: Anorexia nervosa
AN-BP: AN, binge-eating/purging type
AN-R: AN, restricting type
ANOVA: Analysis of variance
ARFID: Avoidant/restrictive food intake disorder
BDI: Beck’s Depression Inventory
BMI: Body mass index
CO: Cooperativeness
DSM-IV-TR: Diagnostic and Statistical Manual of Mental Disorders, 4th edition, text revision
DSM-5: Diagnostic and Statistical Manual of Mental Disorders, 5th edition
ED: Eating disorder
EDNOS: Eating disorder, not otherwise specified
HA: Harm avoidance
NS: Novelty seeking
OSFED: Other specified feeding or eating disorder
PS: Persistence
RD: Reward dependence
SD: Self-directedness
ST: Self-transcendence
TCI: Temperament and character inventory
